# Cardiac Memory T-wave Inversions Noted with Ventricular Pacing: A Possible Electrocardiographic Marker of Appropriate Conduction System Pacing

**DOI:** 10.19102/icrm.2023.14085

**Published:** 2023-08-15

**Authors:** Sergio F. Cossú

**Affiliations:** ^1^Lehigh Valley Health Network, Allentown, PA, USA

**Keywords:** Cardiac memory, conduction system pacing, left bundle branch pacing, memory T-waves

## Abstract

Cardiac memory is a common condition occurring after a period of abnormal depolarization, such as with right ventricular apical pacing. With restoration of normal conduction, the T-wave “remembers” the direction of the QRS vector of the previously aberrantly conducted complexes, creating diffusely inverted T-waves on the electrocardiogram. The presence of diffuse T-wave inversions with this phenomenon may be confused with myocardial ischemia and may continue to be present for several weeks after restoration of normal conduction. Here, an interesting electrocardiogram obtained after pacemaker implantation showing the opposite effect, ie, the finding of memory T-waves occurring during pacing after a period of intrinsic atrioventricular nodal conduction, is presented. In this case, the patient had an underlying left bundle branch block, which subsequently normalized as a result of conduction system pacing. The memory T-waves became evident after pacing was performed, suggesting a potential marker for restoration of the normal ventricular activation sequence with left bundle branch pacing and normalization of the baseline intraventricular conduction defect.

Cardiac memory is an extremely common condition seen in clinical practice. It is usually encountered following a period of abnormal depolarization, such as with right ventricular pacing, intermittent left bundle branch block (LBBB), ventricular tachycardia, or intermittent pre-excitation, as well as following catheter ablation of a manifest accessory pathway.^1–5^ After restoration of normal conduction, the T-wave “remembers” the direction of the QRS vector of the previously aberrantly conducted complexes.^1,6,7^ The abnormal T-wave morphology can at times mimic ischemic changes on the electrocardiogram (ECG), and the clinician must be extremely cognizant of the previous electrocardiographic patterns to account for the current findings.^2^ One very common occurrence is that of memory T-waves becoming evident with the restoration of normal atrioventricular (AV) conduction following a period of right ventricular pacing. An interesting ECG obtained after pacemaker implantation showing the opposite effect, ie, the finding of memory T-waves occurring during conduction system pacing after a period of intrinsic AV nodal conduction in a patient with an underlying LBBB, is presented here.

## Case presentation

**Figure 1:**
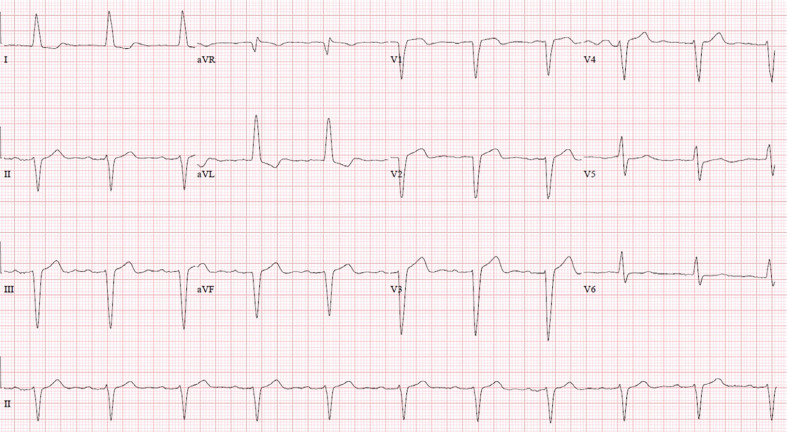
Baseline 12-lead electrocardiogram demonstrating normal sinus rhythm at 63 bpm with an underlying left bundle branch block pattern with an intrinsic QRS duration of 168 ms.

**Figure 2:**
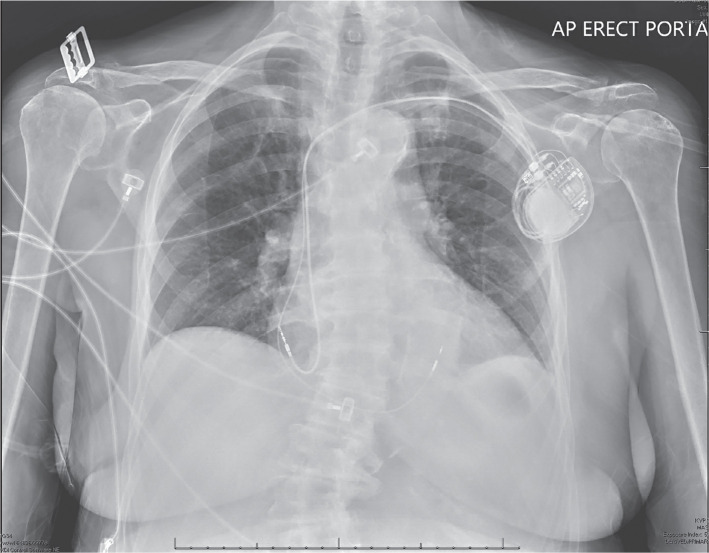
Chest X-ray showing ventricular lead position in the left bundle branch area.

The patient is an 85-year-old woman with a history of hypertension who was admitted with complaints of dizziness and near-syncope. Her baseline ECG demonstrated normal sinus rhythm at 63 bpm with an underlying LBBB pattern with a QRS duration of 168 ms (≥120 ms); broad monophasic R-waves with the absence of a Q-wave in leads I, aVL, V5, and V6; delayed onset of the intrinsicoid deflection; and ST and T-waves in the opposite direction of the QRS in I and aVL **(Figure 1)**. The left ventricular activation time was 88 ms. A 2-dimensional echocardiogram demonstrated a left ventricular ejection fraction of 65% with mild concentric left ventricular hypertrophy and evidence of moderate aortic stenosis. While she was monitored on telemetry, intermittent episodes of Mobitz II AV block were noted to occur and, as such, she was referred for the implantation of a permanent pacemaker with conduction system pacing due to the expectation of a high percentage of ventricular pacing. The patient subsequently underwent implantation of a dual-chamber pacemaker with placement of the ventricular lead (SelectSecure 3830 lead; Medtronic, Minneapolis, MN, USA) in the left bundle region for the purpose of conduction system pacing **(Figure 2)**.

**Figure 3:**
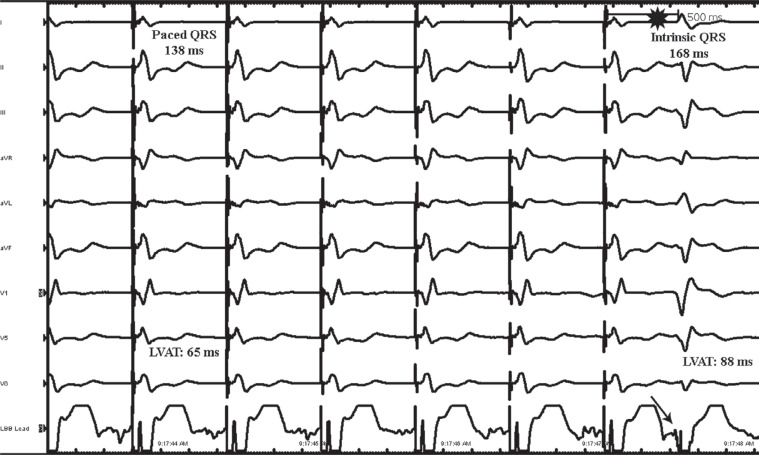
Pacing from the left bundle branch area demonstrating a QRS width of 138 ms with an incomplete right bundle branch pattern noted in lead V1. Leads II, III, and aVF are positive, suggesting a more basal location of the ventricular lead. The left ventricular activation time is 65 ms. *Comparison with the intrinsic QRS complex is seen. The intrinsic QRS demonstrates a QRS duration of 168 ms and a left ventricular activation time of 88 ms. A possible left bundle branch potential is observable from the intracardiac electrogram recorded at this location (arrow). Noted also are inverted T-waves in II, III, aVF, V5, and V6 of the paced complexes in comparison to the intrinsic complex. The axis of these T-waves is in the same direction as the patient’s intrinsic QRS complex in these leads from her baseline electrocardiogram. *Abbreviation:* LVAT, left ventricular activation time.

**Figure 4:**
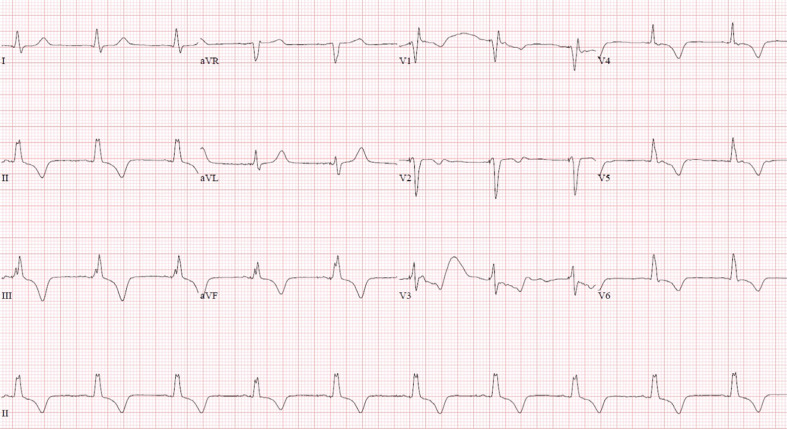
Twelve-lead electrocardiogram immediately following implantation of a left bundle branch pacemaker system. Shown is an atrioventricular sequential paced rhythm at 60 bpm. The QRS width is 138 ms with an incomplete right bundle branch pattern noted in lead V1. Leads II, III, and aVF are positive suggestive of a more basal location of the ventricular lead. Diffuse symmetrical T-wave inversions are noted in the inferior and lateral leads. The axis of these T-waves is in the same direction as the patient’s intrinsic QRS complex in these leads from her baseline electrocardiogram.

**Figure 5:**
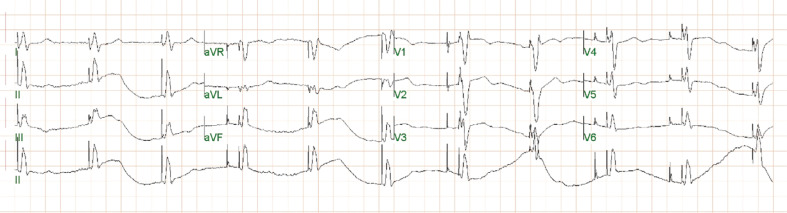
Twelve-lead electrocardiogram obtained 2 weeks following pacemaker implantation. Dual-chamber atrioventricular sequential pacing is noted. Resolution of memory T-waves noted in comparison to **Figure 4**.

Notably, the intraoperative ECG with pacing from the left bundle region in a unipolar configuration demonstrated a paced QRS complex of 138 ms with an incomplete right bundle morphology in V1 **(Figure 3)**. The left ventricular activation time improved to 65 ms. The QRS complex in leads II, III, and aVF was positive, suggestive of a more basal ventricular location of the pacing lead. The electrogram recorded from the lead in this location suggested a possible left bundle potential **(Figure 3)**. Also noted were inverted T-waves in leads II, III, aVF, V5, and V6 in comparison to the intrinsic complex. The measured R-wave from the lead was 18.8 mV with a pacing threshold of 0.5 V at a pulse width of 0.4 ms (unipolar) and an impedance of 893 Ω. The device was programmed in DDDR mode with a lower rate limit of 60 bpm and an upper sensor rate and upper tracking rate of 120 bpm. The sensed AV delay was 130 ms with a paced AV delay of 160 ms. The ventricular output was programmed at 3.5 V at a pulse width of 0.4 ms in a bipolar configuration. An ECG recorded immediately following the procedure demonstrated diffuse symmetrical T-wave inversions, which were noted in the inferior and lateral leads **(Figure 4)**. The axis of these inverted T-waves was in the same direction as the patient’s intrinsic QRS complex in these leads from her baseline ECG **(Figure 1)**. The patient was completely asymptomatic at this point, and there was no reason to suspect underlying ischemia. A follow-up ECG obtained 1 week later demonstrated complete resolution of the T-wave inversions previously noted **(Figure 5)**.

## Discussion

Although there have been several isolated case reports over the years of T-wave changes noted following episodes of tachycardia, the original description of T-wave inversions following pacemaker implantation was presented by Chatterjee et al. in 1969,^8^ who noted the presence of T-wave inversions in the sensed and unpaced ECGs of their patients after a period of pacing. This concept, initially known as the Chatterjee phenomenon, was further explored in 1982 by Rosenbaum, who introduced the concept of cardiac memory and described it as an adaptive mechanism of myocardium repolarization to a new depolarization activation sequence.^6^ The authors noted that it was the change in the activation pattern rather than the change in heart rate that produced the changes in the T-wave direction. They contended that the direction of the T-waves during normal activation will follow the direction of the preceding abnormally activated QRS complexes. During ventricular pacing, there are changes in the repolarization pattern that are not evident due to the marked secondary T-wave abnormalities of ventricular pacing and only become uncovered once pacing is completed and normal conduction continues.^5^ They also speculated that the amplitude of the T-waves will increase the longer the abnormal conduction continues and that repeated episodes of abnormal conduction after normalization of the T-waves will result in more rapid and prominent T-wave changes, which is a phenomenon called accumulation. The lingering effect of cardiac memory has been reported to be present for up to 4 weeks after a period of abnormal depolarization, such as cardiac pacing.^9^

In their study, Chiale et al. were able to demonstrate that brief periods of ventricular pacing will elicit T-wave abnormalities in the same direction as the paced QRS complexes in control patients.^10^ Similarly, in patients with primary T-wave abnormalities or pseudo-primary T-wave abnormalities, such as following catheter ablation of an accessory pathway, pacing from a site that elicited a QRS complex in an opposite direction was able to “normalize” these T-wave abnormalities once pacing was completed. They postulated that these memory T-wave changes occur due to a change in the repolarization sequence that follow the forces of the previously altered depolarization process during ventricular pacing. In doing so, by pacing from different sites, they were able to also “normalize” baseline T-wave abnormalities. These findings emphasize the importance of taking into context the baseline depolarization and repolarization pattern into the development of cardiac memory.^5,10^

Several theories have been proposed for the mechanism behind cardiac memory. One such explanation is due to a change in the transmural repolarization gradient in certain regions of the myocardium with the epicardium being activated earlier, thus resulting in a prolongation of the action potential duration.^4,11^ A decrease in the phase 1 notch of the action potential has been identified in epicardial myocardial cells due to a decrease in the transient outward potassium current (I_to_) in these early activated regions, resulting in a dispersion of the action potential duration transmurally.^11^ Studies have demonstrated that pharmacological blockade of I_to_ will attenuate the development of cardiac memory.^12^ This dispersion of the action potential duration can result in varying degrees of repolarization not only transmurally but also between endocardial ventricular sites.^1,11^ In animal studies, it was shown that, in regions activated early, ie, sites closest to the pacing sites, there was a mild prolongation of the action potential duration, whereas, in regions of late activation, ie, further from the pacing site, there was a significant variation in the action potential duration from a shortening to significant prolongation. Other mechanisms that have been implicated in the development of cardiac memory include angiotensin II–mediated effects on I_to_, alterations in the L-type calcium current (I_Ca_), and rapid activation of the delayed rectifying potassium current (I_Kr_).^11^ In addition, mechanical stretch factors have also been implicated in the development of cardiac memory.^11^

Criteria have been proposed to distinguish memory T-waves from other more potentially malignant electrocardiographic abnormalities, such as myocardial ischemia. The combination of a positive T-wave in aVL, positive or isoelectric T-wave in I, and precordial T-wave inversions greater in the precordial leads than in the inferior leads has been found to be 92% sensitive and 100% specific in differentiating cardiac memory from myocardial ischemia.^13^ Other criteria include (1) a positive T-wave in aVL, (2) a negative or isoelectric T-wave in II, and (3) a negative T-wave in V4–V6 or (4) a corrected QT (QTc) interval < 430 ms.^14^ Although cardiac memory is a relatively benign condition, the QTc has been demonstrated to significantly lengthen, possibly in relation to the ion channel mechanisms described previously.^15^ As a result of this QTc prolongation, cases of pro-arrhythmia have been reported with development of torsade de pointes with the use of anti-arrhythmic agents in this scenario.^16–18^

The typical presentation for cardiac memory is with restoration of normal AV conduction and intrinsic ventricular activation following a period of abnormal activation as with right ventricular apical pacing. Our patient, on the other hand, presented with the development of cardiac memory T-waves with pacing, albeit conduction system pacing. An explanation for this finding is that a “normalized” conduction system had indeed been restored with conduction system pacing. The main finding here is that the patient’s intrinsic QRS complex was of a left bundle morphology with broad monophasic R-waves in I and aVL and T-waves in the opposite direction. The inferior leads on the baseline ECG also demonstrated predominantly negatively directed QRS complexes. With conduction system pacing, activation to the ventricles was now altered, and the ensuing T-waves were thus in the same axis as the original native QRS complexes and showing cardiac memory.

Conduction system pacing, such as with His-bundle pacing or left bundle branch pacing, involves direct stimulation of the cardiac conduction system and would thus result in the improvement of the QRS duration. This could result in a change in the repolarization parameters as well. In a recent case study, Zhong et al. described a similar scenario in which a prolonged period of right ventricular apical pacing followed by implantation of a left bundle branch pacing system also resulted in the development of cardiac memory.^19^ These authors followed up with a subsequent retrospective study in a cohort of 398 patients undergoing placement of left bundle branch pacing leads.^20^ T-wave inversions occurred in 66.3% of patients, which subsequently recovered in 87.8% of them. The leads in which these changes occurred with conduction system pacing varied depending on the type of resting underlying bundle branch block, ie, patients with LBBB tended to have more prominent T-wave inversions in the inferior and anterior leads, whereas patients with right bundle branch block had changes more evident in the high lateral leads. Once again, this emphasizes the importance of the initial depolarization pattern, which relates to the development of cardiac memory once ventricular activation is normalized and the repolarization changes remain. Finally, a recent study demonstrated that conduction system pacing in comparison to biventricular pacing resulted in improved parameters of repolarization as well as resolution of T-wave memory.^21^

Conduction system pacing has recently gained significant popularity in the electrophysiological community as a means to restore and maintain intraventricular synchrony with pacing. Normalization of the QRS complex in a patient with an underlying intraventricular conduction delay could result in the development of cardiac memory. We believe that the electrocardiographic finding in this paper may indeed provide another potential marker in proving the normal ventricular activation sequence with left bundle branch pacing. It is also extremely important to identify this finding and distinguish these changes from other more malignant electrocardiographic changes such as myocardial ischemia.
